# Subthalamic nucleus dynamics track microlesion effect in Parkinson’s disease

**DOI:** 10.3389/fcell.2024.1370287

**Published:** 2024-02-16

**Authors:** Chunkai Peng, Zhuyong Wang, Yujia Sun, Yixiang Mo, Kai Hu, Qingqing Li, Xusheng Hou, Zhiyuan Zhu, Xiaozheng He, Sha Xue, Shizhong Zhang

**Affiliations:** Neurosurgery Center, Department of Functional Neurosurgery, The National Key Clinical Specialty, The Engineering Technology Research Center of Education Ministry of China on Diagnosis and Treatment of Cerebrovascular Disease, Guangdong Provincial Key Laboratory on Brain Function Repair and Regeneration, The Neurosurgery Institute of Guangdong Province, Zhujiang Hospital, Southern Medical University, Guangzhou, China

**Keywords:** Parkinson’s disease, deep brain stimulation, local field potential, beta power, aperiodic components, microlesion effect, subthalamic nucleus

## Abstract

Parkinson’s Disease (PD) is characterized by the temporary alleviation of motor symptoms following electrode implantation (or nucleus destruction), known as the microlesion effect (MLE). Electrophysiological studies have explored different PD stages, but understanding electrophysiological characteristics during the MLE period remains unclear. The objective was to examine the characteristics of local field potential (LFP) signals in the subthalamic nucleus (STN) during the hyperacute period following implantation (within 2 days) and 1 month post-implantation. 15 patients diagnosed with PD were enrolled in this observational study, with seven simultaneous recordings of bilateral STN-LFP signals using wireless sensing technology from an implantable pulse generator. Recordings were made in both on and off medication states over 1 month after implantation. We used a method to parameterize the neuronal power spectrum to separate periodic oscillatory and aperiodic components effectively. Our results showed that beta power exhibited a significant increase in the off medication state 1 month after implantation, compared to the postoperative hyperacute period. Notably, this elevation was effectively attenuated by levodopa administration. Furthermore, both the exponents and offsets displayed a decrease at 1 month postoperatively when compared to the hyperacute postoperative period. Remarkably, levodopa medication exerted a modulatory effect on these aperiodic parameters, restoring them back to levels observed during the hyperacute period. Our findings suggest that both periodic and aperiodic components partially capture distinct electrophysiological characteristics during the MLE. It is crucial to adequately evaluate such discrepancies when exploring the mechanisms of MLE and optimizing adaptive stimulus protocols.

## Introduction

An intriguing phenomenon frequently manifests in patients with Parkinson’s disease (PD) following electrode implantation or nucleus destruction: the patient experiences immediate relief of motor symptoms prior to active stimulation by an impulse pulse generator (IPG). This transient postoperative symptomatic improvement is characterized as the microlesion effect (MLE) (De Cock et al., 2008; Granziera et al., 2008; Maltête et al., 2008; Baumann-Vogel et al., 2017). Current studies on MLE primarily focuses on elucidating its potential formation mechanisms, including the impact of microelectrode penetration during intraoperative microelectrode recording and the edema occurring in the surrounding brain tissue subsequent to electrode implantation, which can disrupt transmission among damaged neuronal synapses and neighboring unaffected neurons (Cersosimo et al., 2009; Chang et al., 2009; Jech et al., 2012; Holiga et al., 2015). However, there remains a lack in the studies regarding the characterization of electrophysiological signals during the period of MLE. The objective of this study was to investigate changes in subthalamic nucleus (STN) local field potential (LFP) during both the postoperative hyperacute period and 1 month post-implantation, with a specific focus on elucidating the electrophysiological characteristics observed during MLE.

Brain oscillations are repetitive patterns of brain activity, and periodic oscillations are associated with many physiological, cognitive, behavioral, and disease states (Buzsáki et al., 2012). However, in addition to periodic oscillations, the power spectrum of brain activity also encompasses aperiodic activity (He, 2014), which was found to correlate strongly with age, cognitive function, and state of consciousness (Voytek et al., 2015; Dave et al., 2018; Colombo et al., 2019; Miskovic et al., 2019; Lendner et al., 2020a; Huang et al., 2020; Tran et al., 2020). Moreover, it is believed that aperiodic activity may contribute to the underlying mechanisms involved in PD (Darmani et al., 2023). Our prior research has demonstrated a noteworthy modulation of scalp electroencephalogram (EEG) activity in patients with PD following the administration of levodopa (Wang et al., 2022). Recent studies have unveiled that deep brain stimulation (DBS) exerts a discernible influence on the aperiodic activity within STN-LFP of PD (Darmani et al., 2022). Recent studies have also found that beta band oscillations of the STN are increased and broadband slopes (aperiodic exponents) are decreased in patients with PD during casual movement (Belova et al., 2021a). Collectively, these findings allude to the potential physiological significance of aperiodic activity in the context of PD. Consequently, another objective of this study is to separate the aperiodic and periodic components of the LFP signal during MLE, seek to comprehensively analyze the distinctive alterations exhibited by these components.

Here, we employ a method of parameterizing neuronal power spectra to separate oscillatory and aperiodic components (Donoghue et al., 2020a), and analyze the signal attributes of STN-LFP during the period of MLE in patients with PD, this investigation serves to enhance understanding of the unique characteristics of neural activity during MLE, thereby contributing to the design and implementation of more precise adaptive deep brain stimulation (aDBS) protocols.

## Materials and methods

### Participants

**FIGURE 1:**
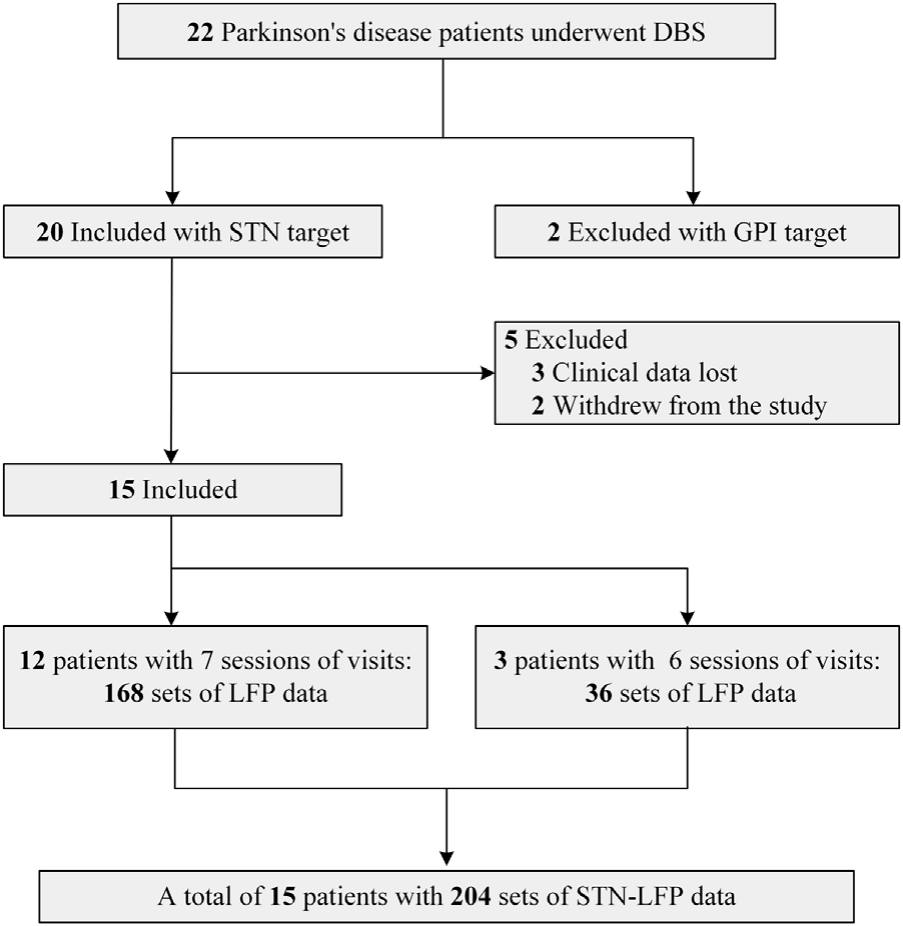
Flow diagram.

This study was a single-center observational study, and the study protocol was approved by the Ethics Committee of Zhujiang Hospital of Southern Medical University (Ethics Approval No. 2021-KY-123). All patients participated in this study after obtaining informed consent and in accordance with the Declaration of Helsinki. Patients were included and excluded from this study according to the following criteria: Inclusion criteria: 1. Diagnosed with primary Parkinson’s disease according to the Chinese Diagnostic Criteria of Parkinson’s Disease (2016 edition) (Parkinson’s Disease and Movement Disorders Group, Chinese Society of Neurology, 2016); 2. Having the indications for DBS surgery as described in the Chinese Expert Consensus on Parkinson’s Disease Deep Brain Electrical Stimulation Therapy (Second edition) (Group of Functional Neurosurgery, Chinese Society of Neurosurgery, 2020); 3. Surgical selection of the bilateral STN nuclei; and Exclusion criteria: 1. Significant cognitive dysfunction (Mini-mental State Examination <24 points); 2. medical coexisting diseases affecting surgery or survival; 3. missing clinical assessment data or unsuccessful recording of STN electrophysiologic signals). The flow diagram depicting patient enrollment is illustrated in Figure 1. Ultimately, a total of 15 patients were included, and the analysis encompassed LFP signals derived from 30 lateral STN nuclei.

### Surgical procedure

Preoperatively, all patients were scanned with a 3.0 T head magnetic resonance imaging (MRI) in axial and coronal positions based on the anterior-posterior commissure (AC-PC) planes, and the scanning sequences were T1-weighted images (layer thickness 1 mm, layer spacing 0 mm), T2-weighted images (layer thickness 2 mm, layer spacing 0 mm), and susceptibility weighted imaging. All anti-Parkinson’s disease medications were discontinued 12 h before implantation. On the day of implantation, a Leksell stereotactic head frame (Elekta Instrument AB, Stockholm, Sweden) was installed under local anesthesia, with the base of the head frame parallel to the plane where the AC-PC was located and with the midline located in the mid-sagittal plane. Cranial Computed tomography (CT) (layer thickness 0.625 mm) was performed after the head frame was installed. Image fusion of MRI and CT images was performed using the Leksell stereotactic system (Elekta Instrument AB, Stockholm, Sweden) to guide the design of STN target coordinates and the optimal trajectory for electrode placement. The bilateral STN-DBS procedure was performed under intravenous and inhalation combined general anesthesia. The location and depth of the STN were confirmed by single-channel microelectrode recording during the procedure, the optimal placement of the DBS leads was verified by intraoperative CT, Surgical complications were excluded, and IPG was performed subcutaneously immediately after confirming the exact lead position.

### Lead localization

**FIGURE 2:**
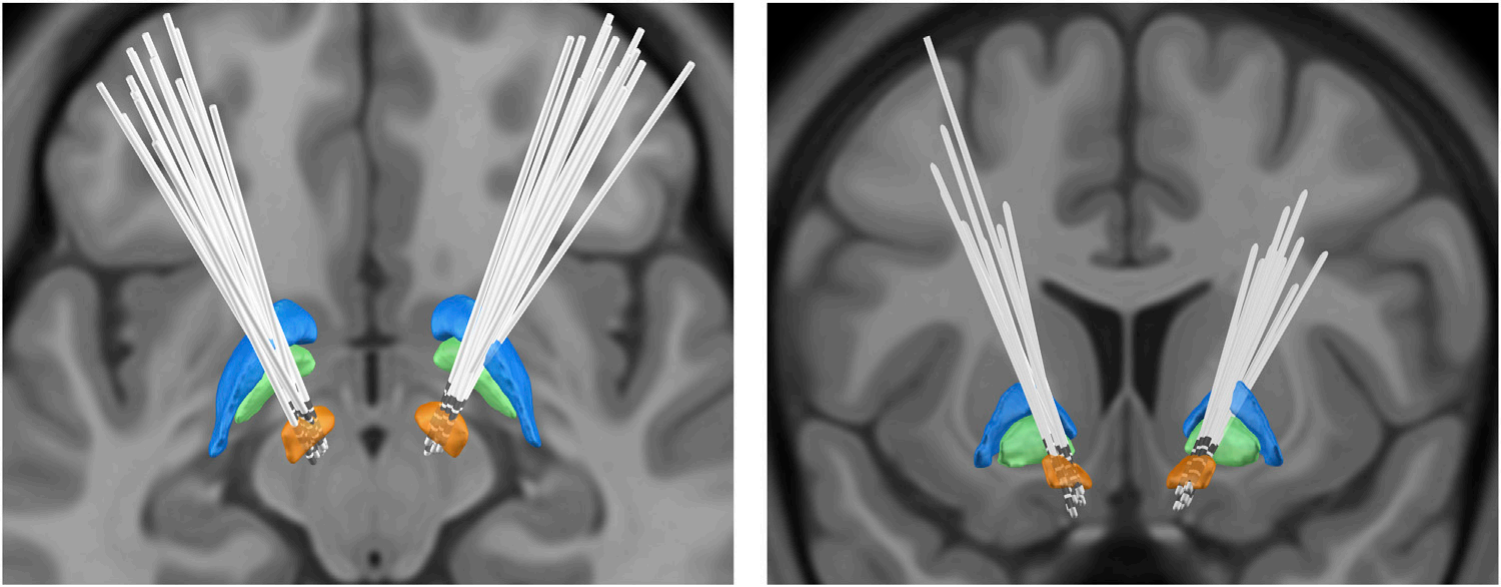
Bilateral deep brain stimulation lead locations for subthalamic nucleus: orange, subthalamic nucleus (STN); green, globus pallidus internus (GPi); blue, globus pallidus externa (GPe).

To confirm the placement of the DBS leads, we used Lead-DBS version 2.3.2 software (https://www.lead-dbs.org/) for postoperative reconstruction localization (Horn and Kühn, 2015; Horn et al., 2017). After correction for inhomogeneity, postoperative thin-layer CT and preoperative MRI images were registered using SPM12 software (https://www.fil.ion.ucl.ac.uk/spm/software/spm12/). Afterward, the postoperative images were non-linearly normalized to the Montreal Neurological Institute (MNI) template brain (International Consortium of Brain Mapping 2009b nonlinear asymmetric) (Avants et al., 2011; Ewert et al., 2019) using the “effective low variance” Advanced Normalization Tools and Symmetric image Normalization approach DBS leads were manually localized following initial, semiautomated trajectory reconstruction. The resultant lead models were warped to MNI space using the aforementioned transforms. The method is consistent with previously reported reconstruction methods (Horn et al., 2017; Darmani et al., 2022). Figure 2 delineates the bilateral STN-DBS lead locations for the entire subject cohort (*n* = 15).

### Experimental protocol

LFP data were collected by an IPG equipped with wireless sensing technology (SR1101, SceneRay, Suzhou, China) (Mandali et al., 2021) postoperatively in the hyperacute period: recording the STN-LFP data of the Medication-OFF state at 6 h, 12 h, 24 h, 36 h, and 48 h postoperatively (Session1-5); One month postoperatively (before active stimulation): STN-LFP signals of Medication-OFF state (Session6) and Medication-ON state (Session7) were recorded.

### Data acquisition

Each DBS electrode has four contacts, the left electrode contacts are numbered as 0, 1, 2, 3 (where contact 0 position is the deepest and contact 3 position is the most superficial), and the right electrode contacts are numbered as 4, 5, 6, 7 (where contact 4 position is the deepest and contact 7 position is the most superficial). For postoperative LFP signals, LFP was obtained from adjacent contact pairs by wireless sensors and digitized by an analog-to-digital converter at a sampling rate of 1,000 Hz. Acquisition criteria: (1) Medication-OFF state at least medication stopping time ≥12 h, and Medication-ON state at least medication taking time ≥1 h; (2) Recording duration was 180 s, and the acquisition contact pairs were Left-STN (0&1, 1&2, 2&3) and Right-STN (4&5, 5&6, 6&7); and (3) The patient was in resting state, with eyes quietly closed and lying flat on the bed.

The LFP data were browsed after acquisition, and records with poor data quality were reacquired. We determined the relative positions of the electrode contacts and the STN by postoperative image fusion reconstruction, and the contact located within the motor sub-region of the STN were included in the analysis. Eventually, a total of 204 LFP spectra were included in the analysis. The relevant data are showed in Supplementary Table S1.

### Data processing

All data preprocessing steps and subsequent analyses were performed using MATLAB (Version 2020a, MathWorks, Inc., Natick, MA, USA) and Python (Version 3.7). First, the local field potential data were bandpass filtered using the pop_firws function in the EEGLAB toolbox (http://www.sccn.ucsd.edu/eeglab/) with lower and upper cutoff frequencies of 1 and 499 Hz, respectively, then apply notch filter at 50 Hz as well as its harmonics (Delorme and Makeig, 2004). The data were then manually inspected for periods of artefact, which were marked for exclusion. Power spectral density (PSD) estimation was performed using the Welch method with a 0.5-s Hamming window, 50% overlap, and a 0.5-Hz resolution. Then, the SpecParam Python package was used to separate periodic and aperiodic components from the spectral results (Donoghue et al., 2020b). The power spectral density $P\left(f\right)$ for each frequency f is expressed as: $P\left(f\right)=L\left(f\right)+\sum_{n}G_{n}\left(f\right)$ . Where the $P\left(f\right)$ is a combination of the aperiodic component $L\left(f\right)$ and Gaussians $G_{n}\left(f\right)$ . The periodic components were parameterized as a mixture of Gaussian distributions: $G_{n}\left(f\right)=\alpha *\text{exp}\left(\frac{-{\left(f-c\right)}^{2}}{2*\omega^{2}}\right)$ . With α as the height of the peak, over and above the aperiodic component, c as the center frequency of the peak, ω as the width of the peak, and f as the array of frequency values. The aperiodic component was also parameterized using a Lorentzian function as: $L\left(f\right)=b-\log\;\left(k+f^{\chi }\right)$ . With b as the broadband “offset,” k as the “knee,” and χ as the ‘exponent’ of the aperiodic fit. The full model was described using these periodic and aperiodic parameters, the goodness of fit was estimated by comparing each fit with the original PSD in terms of the mean absolute error and the $R^{2}$ of the fit. To avoid overfitting, peak_width_limits were set at 1–12, the fitted frequency range was 1–38 Hz, and aperiodic_mode was chosen as “fixed” (fitted without a knee parameter) considering the narrow frequency range. For further analysis, all the models meet the condition that $R^{2}$ value is higher than 0.95. After completing the parameterization of the power spectra, the fitted spectra were used to subtract the aperiodic components to obtain periodic oscillatory components. It is worth noting that the result of the SpecParam algorithm is a logarithmic scale, so we first converted it to a linear scale before subtraction. At last, we use *bandpower* function in MATLAB to calculate the power values, which means calculating the average power in a frequency interval by integrating the power spectral density estimate. We investigated band-limited power of fitted spectra, aperiodic, and periodic components in three frequency bands traditionally used in EEG analysis: theta (4–8 Hz), alpha (8–13 Hz), and beta (13–30 Hz).

### Statistical analysis

Paired *t*-test was used to study the change in motor symptoms in patients preoperatively and 1 month postoperatively under Medication-OFF state, and the effect of levodopa medication on the aperiodic component (exponents and offsets), periodic component (power values) and impedance at 1 month postoperatively. One-way repeated-measure analyses of variance (ANOVA) with Bonferroni’s multiple comparison test was applied to investigate whether the aperiodic components (exponents and offsets), periodic components (power values), and impedance varied with sessions. All statistical analyses were performed with SPSS 25.0 software (SPSS Inc., Chicago, Illinois, USA), and the significance level was set at α = 0.05.

## Results

**FIGURE 3:**
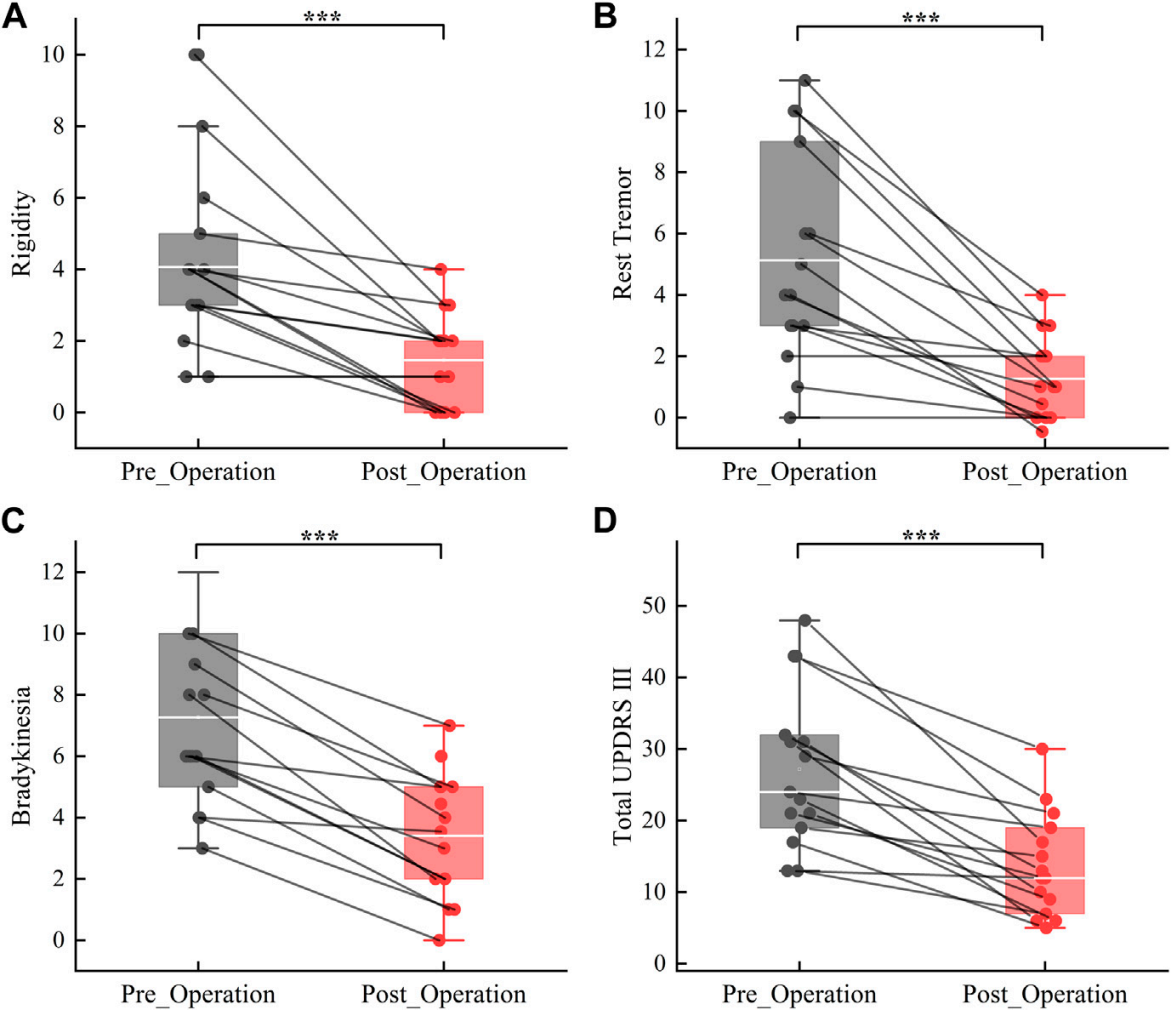
Improvement in motor symptoms from the preoperative to the 1 month postoperativ under Medication-OFF state. Rigidity **(A)**; Rest Tremor **(B)**; Bradykinesia **(C)**; Total UPDRS‐III = Unified Parkinson Disease Rating Scale‐III **(D)**. ***p < 0.001.

Details regarding the preoperative clinical characteristics of patients are given in Table 1. Clinical assessment reveals a significant improvement in motor function at 1 month postoperatively compared to preoperatively under Medication-OFF state (Rigidity: t (14) = 4.80, *p* < 0.001, d = 2.57, 95% CI [1.44; 3.76]; Rest Tremor: t (14) = 5.35, *p* < 0.001, d = 2.86, 95% CI [2.32; 5.42]; Bradykinesia:t (14) = 7.51, *p* < 0.001, d = 4.01, 95% CI [2.76; 4.97]; Total UPDRS III: t (14) = 6.17, *p* < 0.001, d = 3.30, 95% CI [8.83; 18.24]; refer to Figure 3). Subsequently, a comparative analysis was conducted to assess alterations in power values across distinct frequency bands over the course of 7 sequential visitation sessions, and the results are shown below.

**TABLE 1:** Clinical summary of patients included in this study.

Patient	Gender	Disease duration(year)	Age(year)	LEDD(mg)	UPDRS-III preoperative off-medication score
Subject01	M	13	47	480	35
Subject02	F	10	60	800	59
Subject03	M	5	59	780	28
Subject04	F	20	53	375	28
Subject05	F	8	71	750	26
Subject06	M	14	61	600	28
Subject07	F	16	66	385	20
Subject08	F	10	74	625	38
Subject09	M	7	57	375	33
Subject10	N	10	68	425	62
Subject11	F	20	68	280	48
Subject12	M	10	71	500	78
Subject13	M	10	63	375	39
Subject14	F	5	73	950	42
Subject15	M	10	52	900	62

Abbreviations: LEDD, L-dopa equivalent; UPDRS-III, Unified Parkinson’s disease Rating Scale-III.

### Theta power

**FIGURE 4:**
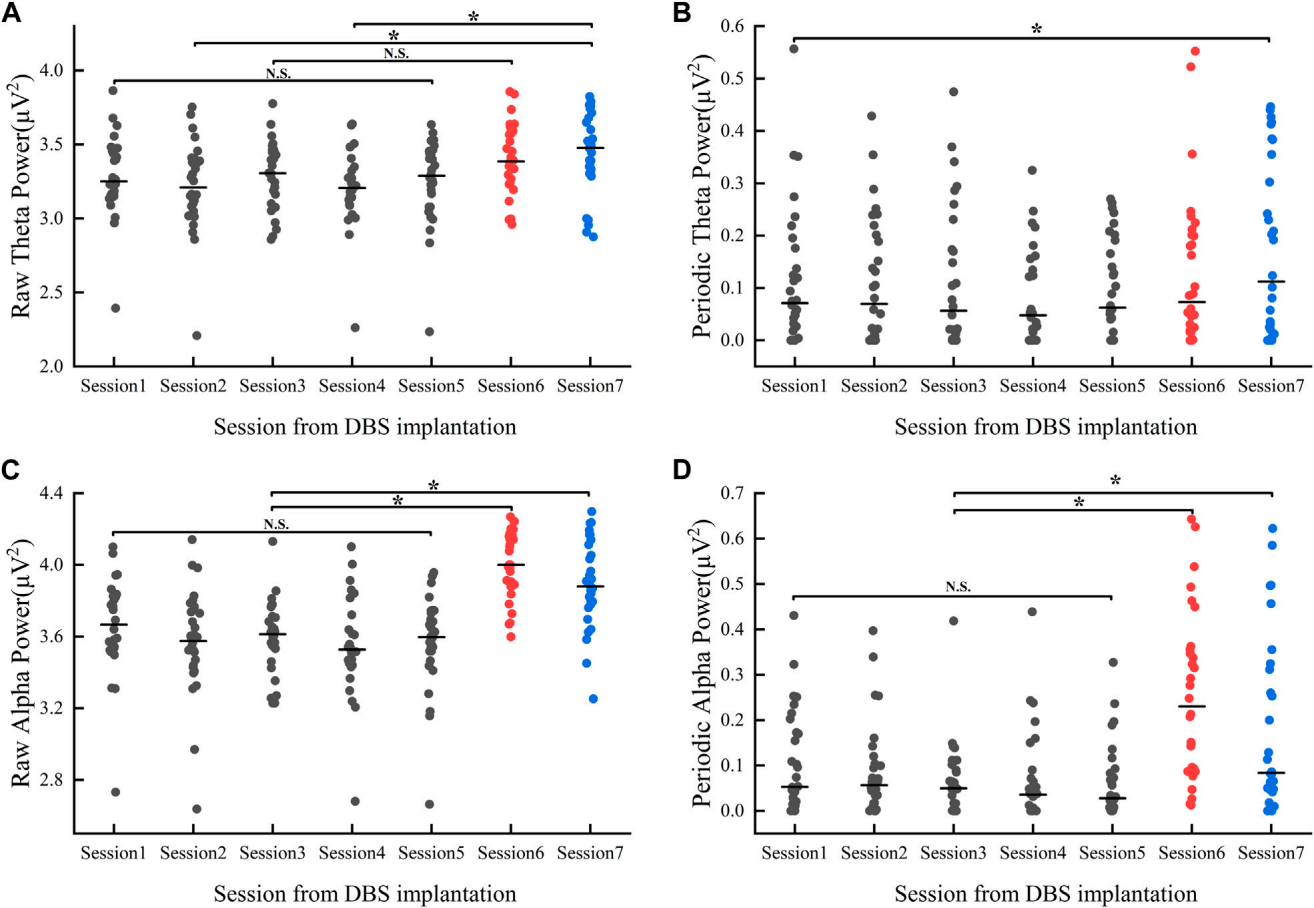
Power value changes in theta **(A, B)** and alpha **(C, D)** band 1 month after implantation: Session1-Session5 represent the power values (gray dots) of the Medication-OFF state at 6 h, 12 h, 24 h, 36 h, and 48 h after implantation. Session6 represents the power values of (red dots) the Medication-OFF state at 1 month after implantation (before active stimulation). Session7 represents the power values (blue dots) of the Medication-ON state 1 month after implantation (before active stimulation). DBS, deep brain stimulation; N.S., statistically not significant; *p < 0.05.

Our results showed that there were within-group differences in raw theta power but not periodic theta power (raw_theta_power: F = 3.431, *p* < 0.01; periodic_theta_power: F = 1.295, *p* > 0.05, refer to Figures 4A, B). In the postoperative hyperacute period, there were no significant differences in both raw and periodic theta power, and in the postoperative 1 month, the state of the levodopa medication also had no significant effect on raw and periodic theta power. However, we found significant differences between the Medication-ON state in 1 month postoperatively and the raw theta power at 12 h postoperatively and 48 h postoperatively.

### Alpha power

Results showed within-group differences in both raw and periodic alpha power (raw_alpha_power: F = 15.587, *p* < 0.01; periodic_alpha_power: F = 9.092, *p* < 0.01, refer to Figures 4C, D). There was no significant difference between raw and periodic alpha power in the postoperative hyperacute period. In the postoperative 1 month, the levodopa Medication-ON or Medication-OFF state had no significant effect on raw and periodic alpha power. Of note, there was a significant difference in both raw and periodic alpha power in the comparison of Medication-ON and Medication-OFF state in the postoperative 1 month and in the postoperative hyperacute period.

### Beta power

**FIGURE 5:**
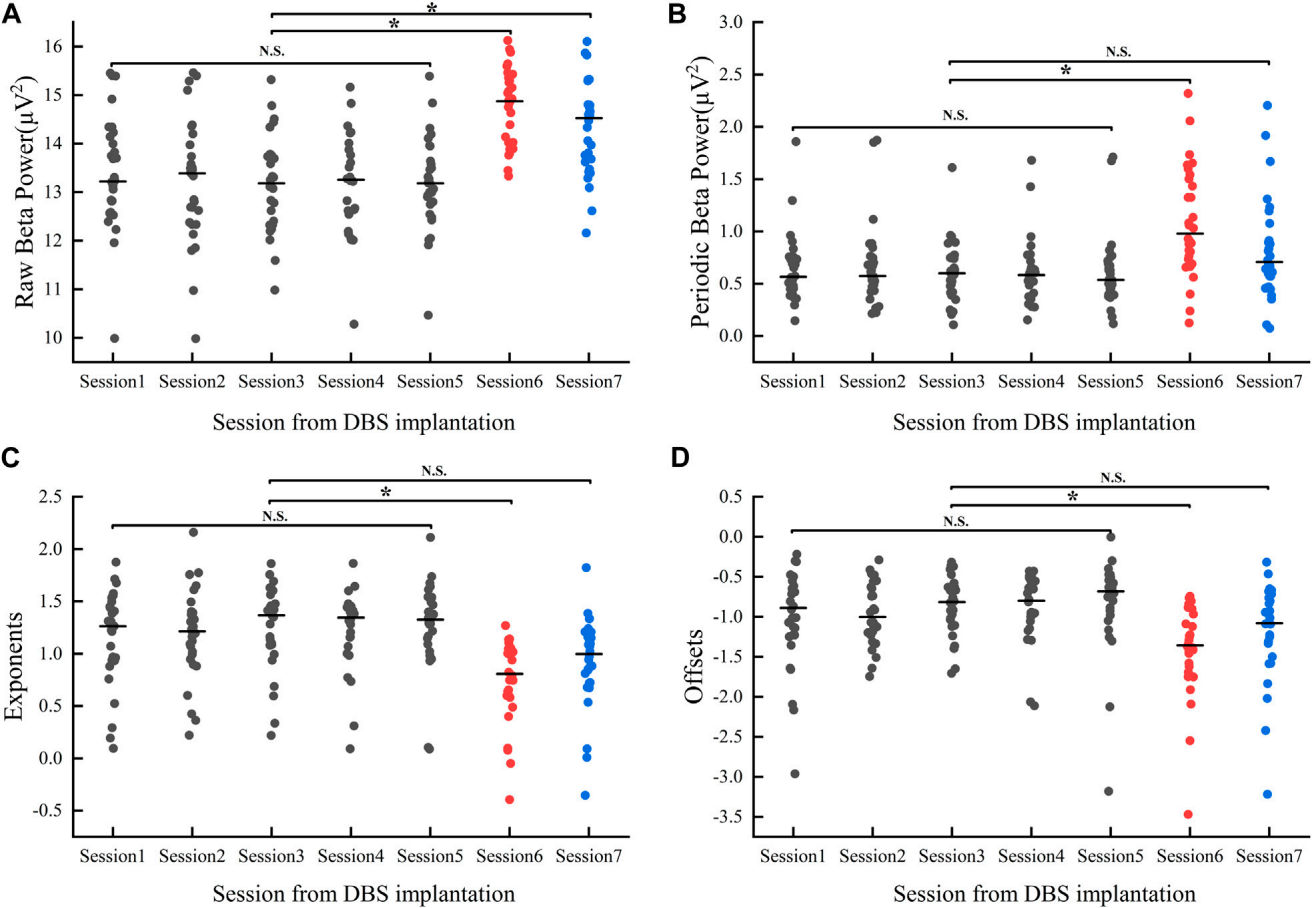
Changes in Beta band power values **(A, B)** and aperiodic parameters **(C, D)** 1 month after implantation: Session1-Session5 represent the power values/aperiodic parameters (gray dots) of the Medication-OFF state at 6 h, 12 h, 24 h, 36 h, and 48 h after implantation. Session6 represents the power values/aperiodic parameters (red dots) of the Medication-OFF state at 1 month after implantation (before active stimulation). Session7 represents the power values/aperiodic parameters (blue dots) of the Medication-ON state at 1 month after implantation (before active stimulation). DBS, deep brain stimulation; NS, statistically not significant; *p < 0.05.

For the beta band, our results showed within-group differences in both raw and periodic beta power (raw_beta_power: F = 12.03, *p* < 0.05; periodic_beta_power: F = 5.85, *p* < 0.05, refer to Figures 5A, B). Multiple comparisons showed no significant differences in both raw and periodic beta power values during the postoperative hyperacute period, whereas significant differences existed between the postoperative hyperacute period and the postoperative 1 month Medication-OFF state. For comparisons between the postoperative hyperacute period and the Medication-ON state, differences were found in raw beta power but not in periodic beta power.

Of note, levodopa medication at 1 month postoperatively effectively reduced beta power values (raw_beta_power: t (29) = 4.78, *p* < 0.05, d = 0.87, 95% CI [0.26; 0.66]; periodic_beta_power: t (29) = 6.06, *p* < 0.05; d = 7.14, 95% CI [0.18; 0.35]), but the above significant differences did not survive correction for multiple comparisons in our study.

### Aperiodic parameters

We compared changes in aperiodic components (exponents, offsets) over time over 7 sessions of visits, and the results showed within-group differences (exponents: F = 6.792, *p* < 0.05; offsets: F = 4.612, *p* < 0.05, refer to Figures 5C, D). Multiple comparison analyses showed no significant differences in either exponent or offset in the postoperative hyperacute period, nor in the comparison of 1 month postoperative Medication-ON state to the postoperative hyperacute period. However, we found a significant difference in the exponents and offsets between the postoperative 1 month Medication-OFF state and the postoperative hyperacute period.

Note that levodopa medication had an effect on aperiodic components at 1 month postoperatively (exponents: t (29) = −7.02, *p* < 0.05, d = −1.28, 95% CI [−0.3; −0.16]; offsets: t (29) = −5.12, *p* < 0.05; d = −0.9, 95% CI [−0.25; −0.1]). Again, the above significant differences did not survive correction for multiple comparisons in our study.

### Impedance

We analyzed impedance in the postoperative hyperacute period and in the postoperative 1 month period and showed that the impedance values in the postoperative 1 month period were significantly higher than in the postoperative hyperacute period and statistically significant differences existed (Impedance: t (29) = −7.4, *p* < 0.05, d = −1.3, 95% CI [−1.1; −0.63]).

## Discussion

In this study, we analyzed the STN-LFP signal characteristics of PD in the post-operative hyperacute period and 1 month after implantation. Here, our results showed that there were significant dynamic changes in power, aperiodic components, and electrode impedance in different frequency bands during the postoperative one-month period. Specifically, we found that beta power, which is closely related to motor symptoms in patients with PD, remained relatively stable in the postoperative hyperacute period, then increased significantly as the MLE waned at the Medication-OFF state in the postoperative 1 month, and then finally declined significantly after levodopa medication treatment. For aperiodic components, exponents and offsets decreased significantly at 1 month postoperatively and were pulled back to the postoperative hyperacute level after medication treatment. Overall, these findings point to contribute further insights into the pathophysiology of PD and to discern how microlesion impact the neural dynamics of interest.

Our results reveal that, 1 month post-implantation, beta power exhibits a significant decrease under the Medication-ON state when compared to the medication OFF state, consistent with prior studies (Belova et al., 2021b; Nie et al., 2021; Chen et al., 2022; Darcy et al., 2022). However, results showed that raw beta power differed significantly between the postoperative hyperacute period and the Medication-ON state, whereas periodic beta power did not differ significantly. For the differences in the above results, we hypothesized that, given the long-neglected physiological significance of aperiodic activity, it may have confounded the estimates of narrow-band power in previous LFP studies, blurring the differences in oscillatory activity between bands and leading to inconsistent conclusions. Therefore, we separated aperiodic activity and compared the differences between raw and periodic power, and the results showed a significant difference between raw power and periodic power at 1 month postoperatively. We suggest that aperiodic components interfere with the identification of oscillatory components to some extent, and that parameterizing neuronal power spectrum might better characterize disease-related electrical activity.

Specifically, the aperiodic components (exponents and offsets) were significantly decreased in the Medication-OFF state 1 month after implantation compared to the postoperative hyperacute period and were pulled back to the postoperative hyperacute period level after administration of levodopa medication. Our previous studies finding that levodopa administration selectively modulates the aperiodic parameters of scalp EEG in PD (Wang et al., 2022), and Ghazaleh et al. finding that the aperiodic parameters of the STN-LFP systematically increased and then stabilized at 6 months after DBS (Darmani et al., 2022), suggest an effect of either drugs or electrical stimulation on aperiodic activities of patients with PD.

Gao et al. have postulated that alterations in spectral exponent are contingent upon global shifts in the excitatory/inhibitory (E/I) balance (Gao et al., 2017). Specifically, a decrease in spectral exponent (flatter spectrum) is indicative of heightened excitatory activity, while an increase in spectral exponent steeper spectrum) suggests an elevation in inhibitory activity. Furthermore, Lendner et al. observed that individuals in an anesthetized state exhibit higher exponent as compared to their awake counterparts (Lendner et al., 2020b), and Yang et al. reported that epileptic patients demonstrate lower exponents at the onset of seizures when contrasted with the preictal period (Yang et al., 2023). These investigations, which focus on disorders associated with the E/I balance, significantly enhance our comprehension of the intricate relationships between spectral indices and the equilibrium of excitatory and inhibitory mechanisms. Therefore, the observed modulatory impact of levodopa medications on aperiodic parameters in our study may signify an augmentation of inhibitory activity. Notably, the heightened index during the hyperacute phase in comparison to the one-month post-implantation interval might indicate higher inhibitory levels. Considering the superior motor performance exhibited during the hyperacute period, enhancing inhibitory activity may emerge as a more favorable target for aDBS modulation.

In addition, we found that impedance increases at 1 month postoperatively, with significant differences compared with the postoperative hyperacute period, which is consistent with the findings of C Lungu et al., who found that electrode impedance decreased slowly within 1 week after implantation and then began to increase, with impedance values peaking at 3 weeks postoperatively and stabilizing over time (Lungu et al., 2014). Notably, the anatomic correlates of electrode impedance and the effect of electrode impedance on neurophysiological data remain unclear, and further studies are needed to illustrate the impact of electrode-tissue interface properties on neurophysiology-related studies.

To date, studies on MLE after implantation in PD patients have focused on the underlying mechanisms of MLE, changes in brain network function, and whether MLE can serve as a predictor of DBS efficacy. The present study is the first research report to describe the characteristics of ultrashort-term LFP signaling after implantation. However, we must recognize that there are some limitations to this study. First, we lacked data on the assessment of clinical symptoms in the postoperative hyperacute period, because it is difficult for patients to complete the assessment during the postoperative hyperacute period. Second, our sample size is relatively small. Subsequent studies could consider including more participants. In the future, we can consider using electrodes with higher density and more contacts to acquire LFP data in STN nucleus tractus subregions for electrophysiological mapping construction of deep brain structures. The realization of these limitations will help guide the direction of future research to deeply explore the mechanism and clinical significance of MLE after DBS in PD patients, and to set programmed directions and goals for future clinical applications of aDBS.

## Conclusion

In summary, our study delved into the distinctive electrophysiological attributes pertaining to the periodic oscillatory and aperiodic components during the hyperacute period and 1 month post-implantation in PD patients. Furthermore, we examined the dynamic alterations exhibited by these components in the one-month post-implantation period and scrutinized the impact of levodopa medication on both facets.

In light of our findings, it becomes evident that comprehending the dissimilarities in the properties of these periodic oscillations and aperiodic components within the MLE window is crucial. These insights should be integral to any future endeavors aimed at elucidating the mechanisms underlying MLE and establishing optimal programmable parameters for aDBS.

## Data Availability

The original contributions presented in the study are included in the article/Supplementary Material, further inquiries can be directed to the corresponding authors.
