# Supplementary material for: Subthalamic nucleus dynamics track microlesion effect in Parkinson’s disease
Source: Front Cell Dev Biol. 2024 Feb 16;12:1370287. doi: 10.3389/fcell.2024.1370287 (PMC10906266; doi:10.3389/fcell.2024.1370287)
Supplement: Supplementary file 1 [file Table1.DOCX]

Supplementary Material

# Supplementary Figures and Tables

# **1.1 Supplementary Table**

**Supplementary Table 1** : The contact of interest for STN-LFP recording.

| Time | Before discharge after surgery | | | | | | | | | | 1 month after surgery | | | |
| --- | --- | --- | --- | --- | --- | --- | --- | --- | --- | --- | --- | --- | --- | --- |
| Session | Post-Surgery | | Post-Surgery | | Post-Surgery | | Post-Surgery | | Post-Surgery | | Medication | | Medication | |
|  | 6h | | 12h | | 24h | | 36h | | 48h | | OFF | | ON | |
| Participant | Left | Right | Left | Right | Left | Right | Left | Right | Left | Right | Left | Right | Left | Right |
| Subject01 | 1-2 | 5-6 | 1-2 | 5-6 | 1-2 | 5-6 | None | None | 1-2 | 5-6 | 1-2 | 5-6 | 1-2 | 5-6 |
| Subject02 | 0-1 | 4-5 | 0-1 | 4-5 | 0-1 | 4-5 | 0-1 | 4-5 | 0-1 | 4-5 | 0-1 | 4-5 | 0-1 | 4-5 |
| Subject03 | 0-1 | 4-5 | 0-1 | 4-5 | 0-1 | 4-5 | 0-1 | 4-5 | 0-1 | 4-5 | 0-1 | 4-5 | 0-1 | 4-5 |
| Subject04 | 1-2 | 4-5 | 1-2 | 4-5 | 1-2 | 4-5 | 1-2 | 4-5 | 1-2 | 4-5 | 1-2 | 4-5 | 1-2 | 4-5 |
| Subject05 | 1-2 | 5-6 | 1-2 | 5-6 | 1-2 | 5-6 | 1-2 | 5-6 | 1-2 | 5-6 | 1-2 | 5-6 | 1-2 | 5-6 |
| Subject06 | 2-3 | 4-5 | 2-3 | 4-5 | 2-3 | 4-5 | 2-3 | 4-5 | 2-3 | 4-5 | 2-3 | 4-5 | 2-3 | 4-5 |
| Subject07 | 0-1 | 4-5 | 0-1 | 4-5 | 0-1 | 4-5 | None | None | 0-1 | 4-5 | 0-1 | 4-5 | 0-1 | 4-5 |
| Subject08 | 0-1 | 5-6 | 0-1 | 5-6 | None | None | 0-1 | 5-6 | 0-1 | 5-6 | 0-1 | 5-6 | 0-1 | 5-6 |
| Subject09 | 2-3 | 5-6 | 2-3 | 5-6 | 2-3 | 5-6 | 2-3 | 5-6 | 2-3 | 5-6 | 2-3 | 5-6 | 2-3 | 5-6 |
| Subject10 | 1-2 | 5-6 | 1-2 | 5-6 | 1-2 | 5-6 | 1-2 | 5-6 | 1-2 | 5-6 | 1-2 | 5-6 | 1-2 | 5-6 |
| Subject11 | 0-1 | 4-5 | 0-1 | 4-5 | 0-1 | 4-5 | 0-1 | 4-5 | 0-1 | 4-5 | 0-1 | 4-5 | 0-1 | 4-5 |
| Subject12 | 1-2 | 5-6 | 1-2 | 5-6 | 1-2 | 5-6 | 1-2 | 5-6 | 1-2 | 5-6 | 1-2 | 5-6 | 1-2 | 5-6 |
| Subject13 | 1-2 | 4-5 | 1-2 | 4-5 | 1-2 | 4-5 | 1-2 | 4-5 | 1-2 | 4-5 | 1-2 | 4-5 | 1-2 | 4-5 |
| Subject14 | 1-2 | 4-5 | 1-2 | 4-5 | 1-2 | 4-5 | 1-2 | 4-5 | 1-2 | 4-5 | 1-2 | 4-5 | 1-2 | 4-5 |
| Subject15 | 0-1 | 4-5 | 0-1 | 4-5 | 0-1 | 4-5 | 0-1 | 4-5 | 0-1 | 4-5 | 0-1 | 4-5 | 0-1 | 4-5 |

None: LFP data with low signal-to-noise ratio were not included in the analysis
